# Machine learning to predict dementia for American Indian and Alaska native peoples: a retrospective cohort study

**DOI:** 10.1016/j.lana.2025.101013

**Published:** 2025-02-13

**Authors:** Kayleen Ports, Jiahui Dai, Kyle Conniff, Maria M. Corrada, Spero M. Manson, Joan O’Connell, Luohua Jiang

**Affiliations:** a Department of Epidemiology & Biostatistics, Joe C. Wen School of Population & Public Health, Susan and Henry Samueli College of Health Sciences, University of California, Irvine, 856 Health Sciences Quad, Irvine, CA 92697-7550, USA; b Department of Statistics, Donald Bren School of Information and Computer Sciences, University of California, Irvine, Bren Hall 2019, Irvine, CA 92697-1250, USA; c Department of Neurology, School of Medicine, University of California, Irvine, 1513 Hewitt Hall, 843 Health Sciences Rd, Irvine, CA 92697, USA; d Centers for American Indian and Alaska Native Health, Colorado School of Public Health, University of Colorado Anschutz Medical Campus, 13055 East 17th Place, Aurora, CO 80045, USA

**Keywords:** American Indian and Alaska Native Peoples, Alzheimer’s disease and related dementia (ADRD), All-cause dementia, LASSO, Machine learning, Risk prediction, XGBoost

## Abstract

**Background:**

Dementia is an increasing concern among American Indian and Alaska Native (AI/AN) communities, yet machine learning models utilizing electronic health record (EHR) data have not been developed or validated for this population. This study aimed to develop a two-year dementia risk prediction model for AI/AN individuals actively using Indian Health Service (IHS) and Tribal health services.

**Methods:**

Seven years of data were obtained from the IHS National Data Warehouse and related EHR databases and divided into a five-year baseline period (FY2007–2011) and a two-year dementia prediction period (FY2012–2013). Four algorithms were assessed: logistic regression, Least Absolute Shrinkage and Selection Operator (LASSO), random forest, and eXtreme Gradient Boosting (XGBoost). Dementia Risk Score (DRS)-based and extended models were developed for each algorithm, with performance evaluated by the area under the receiver operating characteristic curve (AUC).

**Findings:**

The study cohort included 17,398 AI/AN adults aged ≥ 65 years who were dementia-free at baseline, of whom 59.8% were female. Over the two-year follow-up, 611 individuals (3.5%) were diagnosed with incident dementia. Extended models for logistic regression, LASSO, and XGBoost performed comparably: AUCs (95% CI) of 0.83 (0.79, 0.86), 0.83 (0.79, 0.86), and 0.82 (0.79, 0.86). These top-performing models shared 12 of the 15 highest-ranked predictors, with novel predictors including service utilization.

**Interpretation:**

Machine learning algorithms utilizing EHR data can effectively predict two-year dementia risk among AI/AN older adults. These models could aid IHS and Tribal health clinicians in identifying high-risk individuals, facilitating timely interventions and improved care coordination.

**Funding:**

10.13039/100000002NIH.

**Research in context**

**Evidence before this study**

The observed growth in the elderly population in the United States (US) is expected to be accompanied by a significant increase in the prevalence of dementia, including the American Indian and Alaska Native (AI/AN) population. Despite being disproportionately affected by many dementia risk factors, AI/AN individuals remain underrepresented in dementia research. Early identification of those at high risk for dementia is essential for timely diagnosis, intervention, and treatment, underscoring the need for accurate and effective risk prediction models.

To assess the existing evidence prior to this investigation, we conducted a literature search using PubMed and Google Scholar for articles on “machine learning,” “AI/AN,” “dementia,” “ADRD,” “electronic health record (EHR) data,” “risk prediction,” “LASSO,” and “XGBoost,” published before August 2024. While various dementia risk prediction models have been developed, few have combined machine learning algorithms with routinely collected EHR or claims data. These investigations demonstrated the feasibility of using machine learning algorithms to develop dementia risk prediction models. However, no prediction models have been developed or validated to predict the risk of all-cause dementia in AI/AN older adults, leaving a significant gap in this critical area.

**Added value of this study**

This investigation is the first to develop an all-cause dementia risk prediction model specifically for AI/AN individuals. This study also compared the data preprocessing efforts and model performance of three machine learning algorithms with a traditional logistic regression model. Consistent with previous literature, our findings suggest that using machine learning algorithms and EHR data can effectively predict dementia risk. Importantly, this study identified several novel predictors of all-cause dementia that were consistent across algorithms.

**Implications of all the available evidence**

The findings from this investigation will assist Indian Health Service and Tribal health clinicians and providers in identifying AI/AN older adults who are active users of IHS/Tribal services and are at a higher risk of developing all-cause dementia, enabling early diagnosis and intervention. Furthermore, our use of EHR data from a large, geographically diverse sample of AI/AN individuals who used IHS and Tribal services provides a valuable framework for other healthcare systems, particularly those serving resource-limited populations.

## Introduction

The population of older adults aged 65 and older in the United States is rapidly growing.^1^ In line with the general population, the population of older American Indian and Alaska Native (AI/AN) adults is projected to increase nearly three-fold between 2020 and 2060.^1^ With age being a well-established risk factor for dementia, the anticipated accompanying growth of dementia prevalence among AI/AN older adults poses a significant challenge for AI/AN healthcare systems, clinicians, communities, and families.

Many AI/AN peoples access health care through the Indian Health Service (IHS) and Tribal health providers, which face challenges from high morbidity and low socioeconomic status among their patients. Over recent decades, in response to the heavy burden of diabetes among AI/AN peoples, IHS and Tribal health systems have implemented evidence-based, community-driven strategies that have proven effective in diabetes prevention and management. However, these systems are not adequately prepared for the upcoming challenges associated with the anticipated rise in dementia among AI/AN peoples.

Dementia is a leading cause of disability, dependency, and mortality among older adults.^2^ While early diagnosis can offer quality-of-life benefits, including access to dementia-specific services and future planning, dementia remains under-detected and under-managed.^3^ In primary care settings, barriers to timely dementia diagnosis include constraints on face-to-face time between clinicians and patients, lack of timely or adequate follow-up, and lack of physician confidence in diagnosis and/or specialist referral.^4^ These barriers are likely compounded by systematic issues faced within AI/AN healthcare systems, including underfunding, lack of clinic accessibility, understaffed clinics, and a shortage of specialists.^5^ A data-driven model to identify AI/AN individuals at high risk for dementia, who are active users of IHS and Tribal health services, could help address these barriers.

The development of dementia risk prediction models using machine learning algorithms has seen significant growth in recent years. Many existing models have relied on data from the Alzheimer’s Disease Neuroimaging Initiative, cognitive testing, genetic information, and biomarkers.^6^^,^^7^ While these data types can enhance predictive accuracy, their high cost and difficulty of collection limit their practicality in primary care settings. In contrast, electronic health record (EHR) data encompass a wide range of clinical, social, and service use measures with high external validity in real world settings. Given the primary care focus of IHS and Tribal health providers, a dementia prediction model using routinely collected EHR data and machine learning algorithms could serve as a practical and valuable tool for promoting early detection of dementia.

To the best of our knowledge, no models have been developed or validated specifically for predicting dementia risk among AI/AN older adults. The absence of AI/AN representation in the datasets used for model development and validation likely limits their generalizability to this population. In this study, focusing on a diverse sample of active AI/AN IHS and Tribal health service users aged 65 years and older, we aimed to develop a two-year risk prediction model for all-cause, incident dementia using EHR data and machine learning algorithms.

## Methods

### Data source

Approximately 27% (2.6 million) of individuals who self-identify as AI/AN in the US Census receive health care funded by IHS and Tribal health programs, which include hospitals, clinics, and health programs operated by IHS, Tribal organizations, and urban Indian health programs.^8^ This investigation utilized data from the IHS *Improving Health Care Delivery Data Project* (IHS Data Project). The dataset contains health status, service use, and treatment cost information from fiscal years (FY) 2007–2013 for over 640,000 AI/AN peoples, representing nearly 30% of AI/AN peoples who utilize services from IHS, Tribal organizations, and urban Indian health programs across 15 IHS Service Units (sites) throughout the US. These 15 sites had limited urban clinic use during the study period; therefore, we refer to them as IHS and Tribal providers in this investigation. The sample is comparable to the national IHS population in terms of age and sex.

In the IHS Data Project dataset, registration, demographic, IHS and Tribal service data were extracted from the National Data Warehouse, while data for services that were provided by non-IHS or Tribal providers but that were paid for by IHS and Tribal programs were obtained from Purchased and Referred Care services. Additional details about this data source have been published elsewhere.^9^

Project personnel partnered with IHS and Tribal organizations involved in the IHS Data Project through a collaborative network. This network convenes regular meetings of three advisory committees (i.e., Steering, Project Site, and Patient), travels to project sites, and follows a process to obtain approvals from IHS National Institutional Review Board (IRB), Tribal IRBs, Councils, and Tribal Authorities, as well as the university’s IRB (Colorado Multiple IRBs). The IHS National IRB approved a Waiver of HIPAA Authorization and a Waiver of Documentation of Informed Consent because the study involved minimal risk and it was impractical to obtain consent.

### Study design

This retrospective cohort study covered a seven-year period, with a five-year baseline period (FY2007-2011) to collect clinical, demographic, and healthcare utilization data, followed by a two-year prediction window (FY2012-2013) to assess incident dementia. The baseline period served as a washout to differentiate between incident and prevalent dementia, a common approach in EHR studies.^7^ The index date, set as October 1, 2011 (the beginning of FY2012), marked the start of the outcome assessment period.

A two-year prediction window was selected based on data availability and the goal of enabling timely interventions for high-risk individuals. Although dementia’s gradual onset means this timeframe may not capture all early indicators of long-term risk, it focuses on providing actionable predictions for immediate clinical use. To assess the robustness of our findings, we conducted a sensitivity analysis using a one-year prediction window (FY2012).

### Study population

The study cohort included AI/AN individuals aged 65 or older, who were active users of IHS and Tribal health services and had no documented dementia diagnosis by the index date. Individuals were excluded if they had fewer than one healthcare encounter in each of the last three years of the baseline period, if their healthcare encounters over the baseline period were limited to encounters without diagnostic codes, or if they had no healthcare encounters during the outcome assessment period. These inclusion and exclusion criteria were designed to minimize misclassification of both predictors and incident dementia by ensuring that individuals were actively receiving care.

### Candidate predictors

Candidate predictors were prespecified based on existing literature, expert knowledge, and the availability of data in the IHS Data Project dataset. Established predictors included age, sex, diabetes, systolic and diastolic blood pressure, body mass index (BMI), history of stroke or ischemic attack, atrial fibrillation, cardiovascular disease subtypes, tobacco use disorder, alcohol use disorder, depression, mood or anxiety disorders, hearing loss, traumatic brain injury, and the use of anti-hypertensive medications, antidiabetic medications, and cardiovascular disease medications.^6^^,^^7^^,^^10^^,^^11^

Emerging evidence suggests that patients with dementia experience increased healthcare utilization in the years preceding diagnosis.^12^ Therefore, we included emergency room visits, hospital observations, and inpatient admissions as indicators of preclinical dementia-related healthcare needs. Studies have also identified associations between preclinical dementia and reductions in BMI, as well as increased variability in systolic and diastolic blood pressure.^13^^,^^14^ These factors were assessed by calculating the coefficient of variation for each metric over the baseline period. Given the high prevalence of diabetes in AI/AN communities and its association with dementia, we included acute diabetic events as markers of disease severity and instability.^15^ Additionally, we considered healthcare coverage types (Medicaid, Medicare, private insurance) to account for potential disparities in care access and quality. Other comorbidities considered, based on data availability, included cancer, chronic kidney disease, and liver disease.

### Predictor definitions

Comorbid conditions were identified using ICD-9 codes from inpatient and outpatient service utilization records, supplemented by medication data. Sightlines™ DxCG Risk Solutions software, commonly used by private insurers and the federal government to identify chronic conditions, was used to detect baseline histories of cardiovascular disease, malignant cancer, hypertension, chronic kidney disease, hearing loss, mental health conditions, and alcohol and tobacco use disorders. Diabetes was identified using a validated algorithm from national studies, which incorporates diagnostic codes, medication codes, and blood glucose levels.^16^ Acute diabetic complication events were defined as hospital admissions or emergency department visits during the baseline period with a principal discharge diagnosis of hyperglycemia, hypoglycemia, or diabetic ketoacidosis. Stroke or ischemic attack and traumatic brain injury were identified using ICD-9 codes based on prior research (Supplementary Table S1).

Clinical measurements of systolic blood pressure, diastolic blood pressure, and BMI were averaged over the baseline period, and the coefficient of variation was calculated for each measure. Medication use was categorized using the Generic Product Identifier system, a 14-character hierarchical classification system that groups medications based on primary therapeutic use.^17^ Medications of interest included anti-diabetic medications (such as metformin, sulfonylureas, thiazolidinediones, and insulin), anti-hypertensive medications (such as diuretics, beta blockers, calcium channel blockers, angiotensin converting enzyme inhibitors, angiotensin receptor blockers, and other antihypertensive medications), and cardiovascular disease medications (such as statins and other antihyperlipidemic medication). Service utilization was represented by the average number of emergency room visits and hospital observations, as well as the average number of inpatient hospitalizations per fiscal year across the baseline period.

### Assessment of dementia

Individuals were identified as having been diagnosed with incident, all-cause dementia if they had at least one qualifying ICD-9 code in their National Data Warehouse or Purchased and Referred Care inpatient and outpatient service utilization records. ICD-9 codes used for identification of dementia are inclusive of those for Alzheimer’s disease (331.0) as well as vascular (290.40, 290.41, 290.42, 290.43), Lewy body (331.82, 332.0 + 331.0), frontotemporal (331.1, 331.11, 331.19), alcohol-induced (291.2), and other types of dementia (046.11, 046.19, 292.82, 333.4, 290.0, 290.10, 290.11, 290.12, 290.13, 290.20, 290.21, 290.3, 290.9, 294.1, 294.10, 294.11, 294.20, 294.21, 294.8, 331.2, 797) that have been used in previous investigations.^18^

### Modelling approaches

Modeling was performed in stages. First, we developed an age-only logistic regression model to establish a baseline performance, as age is a well-established predictor of dementia. This model served as a reference point for evaluating the added predictive value of other clinical and demographic variables.

For each of the four modeling techniques, we then developed two models: (1) a Dementia Risk Score (DRS)-based model and (2) an extended model. The DRS-based model utilized a targeted set of predictors derived from the DRS, a model initially developed using primary care data from a representative United Kingdom cohort, aged 60 to 95, through the Health Improvement Network.^19^ The DRS is one of the few dementia prediction models based on EHR data that has been externally validated across diverse racial and ethnic groups.^7^^,^^20^ However, some variables, such as the social deprivation score, were unavailable in the IHS dataset, limiting its full external validation.

The extended model incorporated additional predictors based on literature and clinical insights, allowing for a more comprehensive set of dementia-related factors beyond those included in the DRS. This expansion allowed us to assess whether incorporating a broader range of predictors would improve model performance, particularly with ensemble methods like random forest and XGBoost, which are well-suited to handle high-dimensional data.

### Modelling algorithms

Four algorithms commonly used in the field of dementia prediction were employed: logistic regression with backward stepwise selection, Least Absolute Shrinkage Operator (LASSO) regression, random forest, and eXtreme Gradient Boosting (XGBoost). Logistic regression served as a benchmark model, valued for its interpretability and ability to provide clear insights into the influence of each predictor. However, logistic regression is constrained by strict statistical assumptions and is less suited for handling high-dimensional data, multicollinearity, sparsity, and missing data.^21^ LASSO, an extension of logistic regression, mitigates some of these limitations by incorporating variable selection and regularization, which improves its suitability for high-dimensional data and reduces overfitting, although it still shares logistic regression’s underlying statistical assumptions and sensitivity to multicollinearity.^21^ Random forest and XGBoost, tree-based ensemble methods, are designed to capture complex, non-linear relationships and interactions, which may be missed by linear models. XGBoost, in particular, requires minimal data preprocessing and often achieves higher predictive accuracy through gradient boosting, an iterative process that enhances precision.^22^ However, tree-based methods generally offer lower interpretability than traditional statistical models. Additional details on each algorithm type are available in the Supplementary Methods.

### Data pre-processing

Model-specific pre-processing steps are outlined in Table 1. For logistic regression and LASSO, scatter plots of each continuous predictor against the logit values were visually inspected to assess linearity. Nonlinear relationships were addressed by categorizing predictors when necessary. Since random forest and XGBoost are not constrained by linearity assumptions, no categorization was required for these models. To prevent overfitting, inaccurate feature importance, and high variance, sparse features were removed in logistic regression and LASSO, while random forest and XGBoost naturally accommodate sparse data. Standardization (mean of zero, standard deviation of one) was applied to LASSO features to ensure comparability of coefficients under penalization.

**Table 1:** Candidate predictors and pre-processing by model type.

Candidate predictor	LR/LASSO^a^ modeling	RF/XGBoost modeling
Age	Continuous	Continuous
Body mass index (BMI)		
Average	Categorical^b^: < 18.5, 18.5 to < 25, 25 to < 30, ≥ 30, Missing^c^	Continuous
Coefficient of variation	Categorical^b^: tertiles and Missing^c^	Continuous
Systolic blood pressure		
Average	Categorical^b^^d^: <120, 120–129, 130–139, and ≥ 140 mmHg	Continuous
Coefficient of variation	Categorical^b^^d^: tertiles	Continuous
Diastolic Blood Pressure		
Average	Categorical^b^^d^: quartiles	Continuous
Coefficient of variation	Categorical^b^^d^: tertiles	Continuous
Emergency room visits and hospital observations	Categorized^b^: <1, 1–2, >2	Continuous
Inpatient hospitalizations	Categorical^b^: 0, 0–1, >1	Continuous
Health care coverage		
Private	Yes/No	Yes/No
Medicaid	Yes/No	Yes/No
Medicare	Omitted^e^	Yes/No
IHS only	Omitted^e^	Yes/No
Sex	Male/Female	Male/Female
Cardiovascular conditions/events		
Stroke or Ischemic attack	Yes/No	Yes/No
Atrial fibrillation	Yes/No	Yes/No
Cardiovascular disease subtype		
Cerebrovascular disease	Yes/No	Yes/No
Ischemic heart disease	Yes/No	Yes/No
Vascular disease	Yes/No	Yes/No
Congestive heart failure/other	Yes/No	Yes/No
Other comorbidities		
Diabetes	Yes/No	Yes/No
Hypertension	Yes/No	Yes/No
Cancer (All)	Yes/No	Yes/No
Chronic kidney disease	Yes/No	Yes/No
Traumatic brain injury	Yes/No	Yes/No
Hearing loss	Yes/No	Yes/No
Mental health diagnoses		
Depression	Yes/No	Yes/No
Mood/anxiety disorder	Yes/No	Yes/No
Substance use disorder		
Alcohol use disorder	Yes/No	Yes/No
Tobacco use disorder	Yes/No	Yes/No
Antidiabetic medications		
Metformin	Yes/No	Yes/No
Sulfonylureas	Yes/No	Yes/No
Thiazolidinediones	Yes/No	Yes/No
Insulin	Yes/No	Yes/No
Anti-hypertensive medications	Yes/No	Yes/No
Cardiovascular disease medications	Yes/No	Yes/No
Severe acute diabetic event	Yes/No	Yes/No

LR: Logistic Regression; LASSO: Least Absolute Shrinkage Operator; RF: Random Forest, XGBoost: Extreme Gradient Boosting.

a All predictors were standardized prior to entry into LASSO algorithm.

b Continuous variable categorized due to evidence of non-linearity with the predictor and the log-odds of dementia.

c Missing category was created when predictor missingness was greater than 5%.

d k nearest neighbor (kNN) imputation used when predictor missingness was less than 5%.

e Omission due to low feature variance and unstable estimates in LR and LASSO.

To address missing data, model-specific approaches were used. XGBoost’s sparsity-aware split-finding algorithm handled missing data internally, while logistic regression and LASSO required imputation before model training. For predictors with less than 5% missing data, a commonly used threshold,^23^ k nearest neighbor imputation was applied to fill gaps based on similar cases. For predictors with more than 5% missing data, we categorized missing values separately.

### Model building and internal validation

**Fig. 1:**
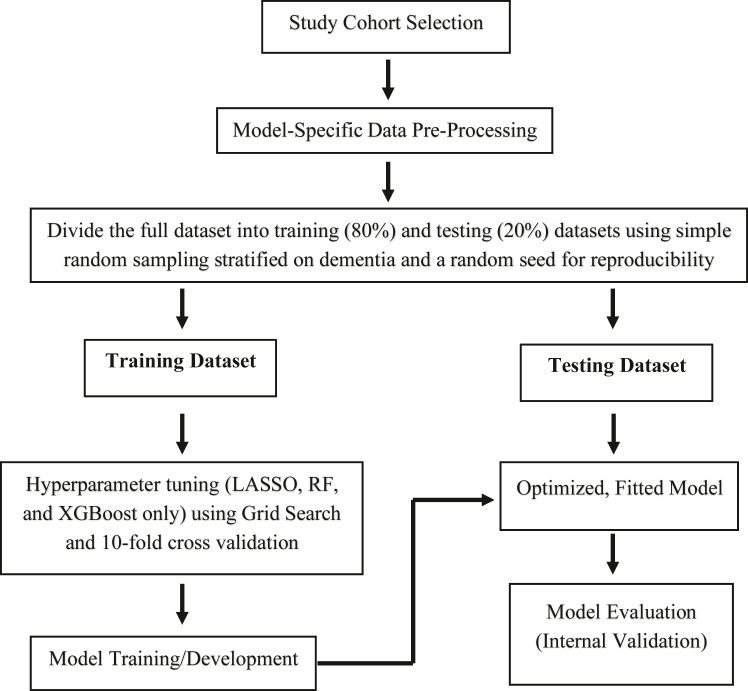
**Dementia prediction model development and validation workflow.** Abbreviations: LASSO: Least Absolute Shrinkage Operator; RF: Random Forest; XGBoost: Extreme Gradient Boosting.

The full study sample was split into training (80%) and testing (20%) datasets through simple random sampling, stratified by incident dementia status. The training dataset was used for hyperparameter tuning and model training, while the testing dataset was reserved for internal validation to assess the performance of each model. Fig. 1 illustrates the overall workflow for model building and validation.

Before training the LASSO, random forest, and XGBoost models, hyperparameter tuning was performed using grid search. A semi-random grid of 50 hyperparameter combinations, generated through Latin Hypercube Sampling, was evaluated with 10-fold cross-validation. The optimal hyperparameter values were selected based on the highest area under the receiver operating characteristic curve (AUC). Each model was then trained on the processed training data using these optimized hyperparameter values.

The predictive performance of each model was evaluated on the testing dataset and compared based on AUC. To evaluate clinical applicability, sensitivity and specificity were examined across a range of thresholds, given the imbalanced nature of the dataset, with dementia being a rare outcome. To ensure robust performance estimates, 95% confidence intervals (CIs) for AUC, sensitivity, and specificity were calculated using bootstrap resampling with 200 iterations.

### Feature importance

Feature importance is quantified differently for each machine learning algorithm. In this investigation, we used the *vip* package in R software (version 4.2.0) to calculate and visualize feature importance for the top-performing models.^24^^,^^25^ For logistic regression and LASSO, feature importance was assessed based on the magnitude of the coefficients and their associated z-statistics, reflecting the statistical significance of each predictor. In random forest, feature importance was determined by impurity reduction, which indicates each feature’s contribution to reducing node impurity and improving prediction accuracy. For XGBoost, feature importance was based on Gain, which quantifies the impact of each feature on improving the model’s objective function. We compared the top 15 variables most predictive of dementia across all algorithms.

### Role of the funding source

The study sponsors were not involved in the design of the study; the collection, analysis, and interpretation of data; the writing of the report; or the decision to submit the paper for publication.

## Results

### Characteristics of excluded participants

**Fig. 2:**
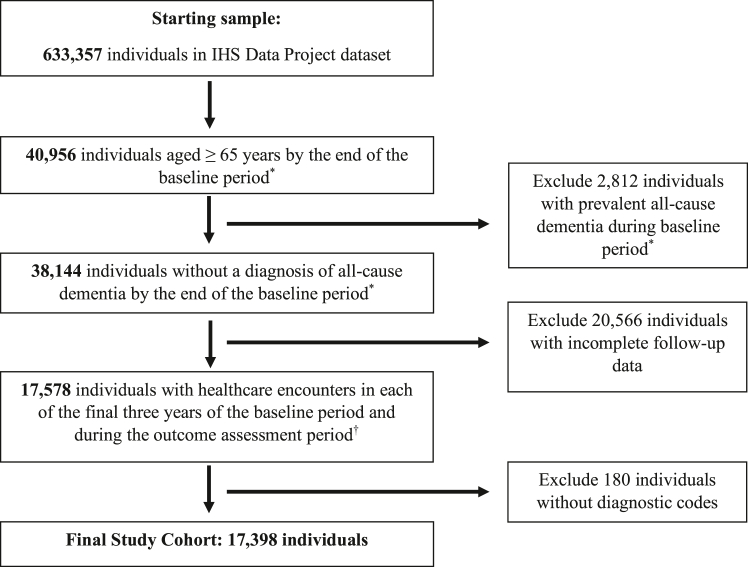
**Flowchart of study cohort identification from IHS Data Project dataset for the development and internal validation of a two-year all-cause dementia prediction model.** ∗Five-year baseline period defined from fiscal year FY2007-2011. ^†^Two-year outcome assessment period defined from FY2012-2013.

The IHS Data Project dataset initially included 38,144 individuals aged 65 years and older who were dementia-free at the index date. Of these, 20,746 individuals (54.4%) were excluded due to incomplete follow-up according to the specified inclusion criteria, resulting in a final study cohort of 17,398 individuals (Fig. 2).

Supplementary Table S2 compares the baseline characteristics of the included and excluded cohorts. Individuals in the included cohort were more likely to have Medicare coverage (97.5% vs. 47.0%) and less likely to be uninsured (2.0% vs. 4.2%), although Medicare coverage data were missing for 48.2% of individuals in the excluded cohort. Additionally, the included cohort had a higher prevalence of comorbid conditions, including obesity (42.3% vs. 17.2%), hypertension (85.2% vs. 48.4%), diabetes (47.5% vs. 23.7%), and congestive heart failure (37.1% vs. 22.1%).

### Study population

Among the 17,398 adults aged 65 years and older, the mean (SD) age was 73.1 (6.3) years, and 10,409 (59.8%) were women. During the two-year follow-up, 611 (3.5%) individuals were diagnosed with incident all-cause dementia.

Those diagnosed with dementia were older, with a mean (SD) age of 79.1 (7.5) years, compared to 72.9 (6.1) years in the non-dementia group. Additionally, the dementia cohort had a higher proportion of individuals with Medicaid coverage (25.2% vs. 13.1%) and a greater prevalence of cardiovascular conditions, including cerebrovascular disease (22.8% vs. 11.5%), stroke or ischemic attack (12.3% vs. 6.2%), and atrial fibrillation (12.6% vs. 6.5%). A detailed comparison of baseline characteristics is provided in Table 2.

**Table 2:** Baseline clinical and demographic characteristics of the study cohort by incident all-cause dementia status.

	No dementia N = 16,787 N (%)	Incident dementia N = 611 N (%)	Full cohort N = 17,398 N (%)
Age			
Mean (SD)	72.9 (6.1)	79.1 (7.5)	73.1 (6.3)
Range	(65.0–103.0)	(65.2–99.0)	(65.0–103.0)
Sex			
Female	10,027 (59.7)	382 (62.5)	10,409 (59.8)
Insurance coverage			
Private	2867 (17.1)	75 (12.3)	2942 (16.9)
Medicaid	2200 (13.1)	154 (25.2)	2354 (13.5)
Medicare	16,364 (97.5)	595 (97.4)	16,959 (97.5)
None	331 (2.0)	15 (2.5)	346 (2.0)
Body mass index (BMI)^a^			
Underweight	105 (0.6)	8 (1.3)	113 (0.7)
Normal weight	2855 (17.0)	160 (26.2)	3015 (17.3)
Overweight	5719 (34.1)	233 (38.1)	5952 (34.2)
Obese	7194 (42.9)	164 (26.8)	7358 (42.3)
Missing	914 (5.4)	46 (7.5)	960 (5.5)
Blood pressure^b^			
Normal	1542 (9.2)	56 (9.2)	1598 (9.2)
Elevated	3887 (23.2)	153 (25.0)	4040 (23.2)
High	11,085 (66.0)	399 (65.3)	11,484 (66.0)
Missing	273 (1.6)	3 (0.5)	276 (1.6)
Cardiovascular disease subtypes^c^			
Cerebrovascular disease	1930 (11.5)	139 (22.8)	2069 (11.9)
Ischemic heart disease	4359 (26.0)	201 (32.9)	4560 (26.2)
Vascular disease	4939 (29.4)	257 (42.1)	5196 (29.9)
Congestive heart failure/other	6157 (36.7)	305 (49.9)	6462 (37.1)
Cardiovascular conditions/events^c^			
Stroke or Ischemic attack	1046 (6.2)	75 (12.3)	1121 (6.4)
Atrial fibrillation	1092 (6.5)	77 (12.6)	1169 (6.7)
Other comorbidities^c^			
Diabetes	7943 (47.3)	321 (52.5)	8264 (47.5)
Hypertension	14,289 (85.1)	540 (88.4)	14,829 (85.2)
Cancer (All)	1660 (9.9)	69 (11.3)	1729 (9.9)
Chronic kidney disease	3216 (19.2)	168 (27.5)	3384 (19.5)
Traumatic brain injury	414 (2.5)	28 (4.6)	442 (2.5)
Hearing loss	2674 (15.9)	170 (27.8)	2844 (16.4)
Mental health diagnoses^c^			
Depression	3626 (21.6)	168 (27.5)	3794 (21.8)
Mood/anxiety disorder (excluding depression)	914 (5.4)	45 (7.4)	959 (5.5)
Substance use disorder^c^			
Alcohol use disorder	801 (4.8)	61 (10.0)	862 (5.0)
Tobacco use disorder	2549 (15.2)	70 (11.5)	2619 (15.1)
Antidiabetic medications			
Metformin	4992 (29.7)	162 (26.5)	5154 (29.6)
Sulfonylureas	3905 (23.3)	147 (24.1)	4052 (23.3)
Thiazolidinediones	3313 (19.7)	113 (18.5)	3426 (19.7)
Insulin	2627 (15.7)	125 (20.5)	2752 (15.8)
Anti-hypertensive medications^d^	14,177 (84.5)	537 (87.9)	14,714 (84.6)
Cardiovascular disease medications^e^	10,674 (63.6)	356 (58.3)	11,030 (63.4)
Average inpatient hospitalizations per fiscal year			
0	12,482 (74.4)	326 (53.4)	12,808 (73.6)
>0 to <1	3935 (23.4)	236 (38.6)	4171 (24.0)
≥1	370 (2.2)	49 (8.0)	419 (2.4)
Average emergency room visits or hospital observations per fiscal year			
<1	13,697 (81.6)	389 (63.7)	14,086 (81.0)
≥1 to <2	1979 (11.8)	124 (20.3)	2103 (12.1)
≥2	1111 (6.6)	98 (16.0)	1209 (7.0)
Severe acute diabetic event	552 (3.3)	52 (8.5)	604 (3.5)

a Average BMI across baseline period with WHO algorithm for BMI categorization. Underweight: BMI < 18.5; Normal Weight: BMI 18.5 to 25; Overweight: 25 < BMI < 30; Obese: BMI ≥ 30.

b Average blood pressure across baseline period with the following definitions: Normal: Systolic Blood Pressure (SBP) < 120 mmHg and Diastolic Blood Pressure (DBP) < 80 mmHg; Elevated: 120 mmHg ≤ SBP < 130 mmHg and DBP < 80 mmHg; High: SBP ≥ 130 mmHg or DBP ≥ 80 mmHg.

c Comorbidities present in electronic health records during the baseline period.

d Anti-hypertensive medications included diuretics, beta blockers, calcium channel blockers, angiotensin converting enzyme inhibitors, angiotensin receptor blockers, and other anti-hypertensive medications.

e Cardiovascular disease medications included statins and antihyperlipidemic medications.

### Model performance

The optimized hyperparameter values for each model are provided in Supplementary Table S3. Table 3 presents the discriminative performance of each model on the testing dataset, as evaluated by AUC. The age-only logistic regression model achieved an AUC of 0.76 (95% CI: 0.71, 0.80). The DRS-based models demonstrated generally comparable performance, with AUCs of 0.80 (95% CI: 0.77, 0.83) for logistic regression, 0.80 (95% CI: 0.77, 0.83) for LASSO, 0.76 (95% CI: 0.73, 0.80) for random forest, and 0.80 (95% CI: 0.75, 0.84) for XGBoost.

**Table 3:** Model performance on the testing dataset, measured by area under the receiver operator characteristic curve (AUC).

Model	AUC (95% CI)
LR (age-only)	0.76 (0.71, 0.80)
LR (DRS-based)	0.80 (0.77, 0.83)
LR (extended)	0.83 (0.79, 0.86)
LASSO (DRS-based)	0.80 (0.77, 0.83)
LASSO (extended)	0.83 (0.79, 0.86)
RF (DRS-based)	0.76 (0.73, 0.80)
RF (extended)	0.76 (0.72, 0.81)
XGBoost (DRS-based)	0.80 (0.75, 0.84)
XGBoost (extended)	0.82 (0.79, 0.86)

LR: Logistic Regression; LASSO: Least Absolute Shrinkage Operator; RF: Random Forest; XGBoost: Extreme Gradient Boosting; DRS: Dementia Risk Score; CI: Confidence Interval.

**Fig. 3:**
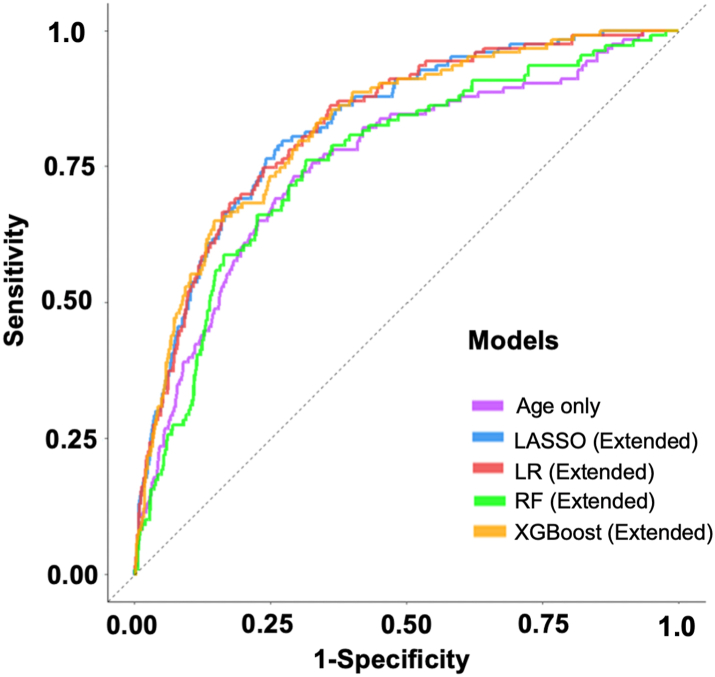
**Receiver operatingcharacteristic curves for the age-only, LASSO (extended), LR (extended), RF (extended) and XGBoost (extended) two-year incident, all-cause dementia prediction models∗.** Abbreviations: LR: Logistic Regression; LASSO: Least Absolute Shrinkage Operator; RF: Random Forest; XGBoost: Extreme Gradient Boosting.

The inclusion of additional candidate predictors in the extended models improved discriminatory performance across most models. The extended logistic regression, LASSO, and XGBoost models were the top performers, with AUCs of 0.83 (95% CI: 0.79, 0.86), 0.83 (95% CI: 0.79, 0.86), and 0.82 (95% CI: 0.79, 0.86), respectively. In contrast, the extended random forest model did not demonstrate an improved performance over the DRS-based model, with an AUC of 0.76 (95% CI: 0.72–0.81). Receiver operating characteristic (ROC) curves for the age-only, extended logistic regression, LASSO, random forest, and XGBoost models are shown in Fig. 3.

In the sensitivity analysis with a 1-year follow-up window, the AUCs (95% CI) for the top-performing extended logistic regression, LASSO, and XGBoost models were 0.79 (0.57, 0.80), 0.79 (0.56, 0.80) and 0.79 (0.68, 0.83), respectively (Supplementary Table S4).

Sensitivity and specificity for the top-performing models were largely consistent (Table 4). At a 2% classification threshold, XGBoost demonstrated a sensitivity of 90% (95% CI: 86%, 95%) and a specificity of 53% (95% CI: 51%, 54%), while LASSO had a sensitivity of 89% (95% CI: 83%, 94%), and a specificity of 52% (95% CI: 51%, 54%). At a higher threshold of 4%, where both models showed decreasing sensitivity and increasing specificity, XGBoost achieved 73% sensitivity (95% CI: 65%, 80%) and 75% specificity (95% CI: 74%, 76%), while the LASSO showed a sensitivity of 74% (95% CI: 68%, 83%) and a specificity of 77% (95% CI: 75%, 78%).

**Table 4:** Sensitivity and specificity of top-performing dementia risk models at various threshold values on the testing dataset.

Model	Threshold	Sensitivity (%) (95% CI)	Specificity (%) (95% CI)
LR (Extended)			
	0.010	97% (93%, 99%)	28% (27%, 30%)
	0.020	89% (84%, 94%)	55% (54%, 57%)
	0.025	86% (81%, 91%)	64% (62%, 65%)
	0.030	81% (74%, 86%)	69% (67%, 71%)
	0.040	72% (64%, 79%)	77% (76%, 79%)
	0.050	68% (60%, 75%)	83% (82%, 84%)
LASSO (Extended)			
	0.010	98% (97%, 100%)	20% (19%, 21%)
	0.020	89% (83%, 94%)	52% (51%, 54%)
	0.025	86% (80%, 92%)	61% (59%, 62%)
	0.030	81% (75%, 88%)	67% (65%, 69%)
	0.040	74% (68%, 83%)	77% (75%, 78%)
	0.050	67% (59%, 75%)	82% (81%, 84%)
XGBoost (Extended)			
	0.010	99% (96%, 100%)	19% (18%, 21%)
	0.020	90% (86%, 95%)	53% (51%, 54%)
	0.025	86% (80%, 91%)	61% (59%, 62%)
	0.030	83% (76%, 88%)	67% (65%, 68%)
	0.040	73% (65%, 80%)	75% (74%, 76%)
	0.050	68% (58%, 75%)	81% (80%, 82%)

LR: Logistic Regression; LASSO: Least Absolute Shrinkage Operator; XGBoost: Extreme Gradient Boosting; CI: Confidence Interval.

### Feature importance

**Fig. 4:**
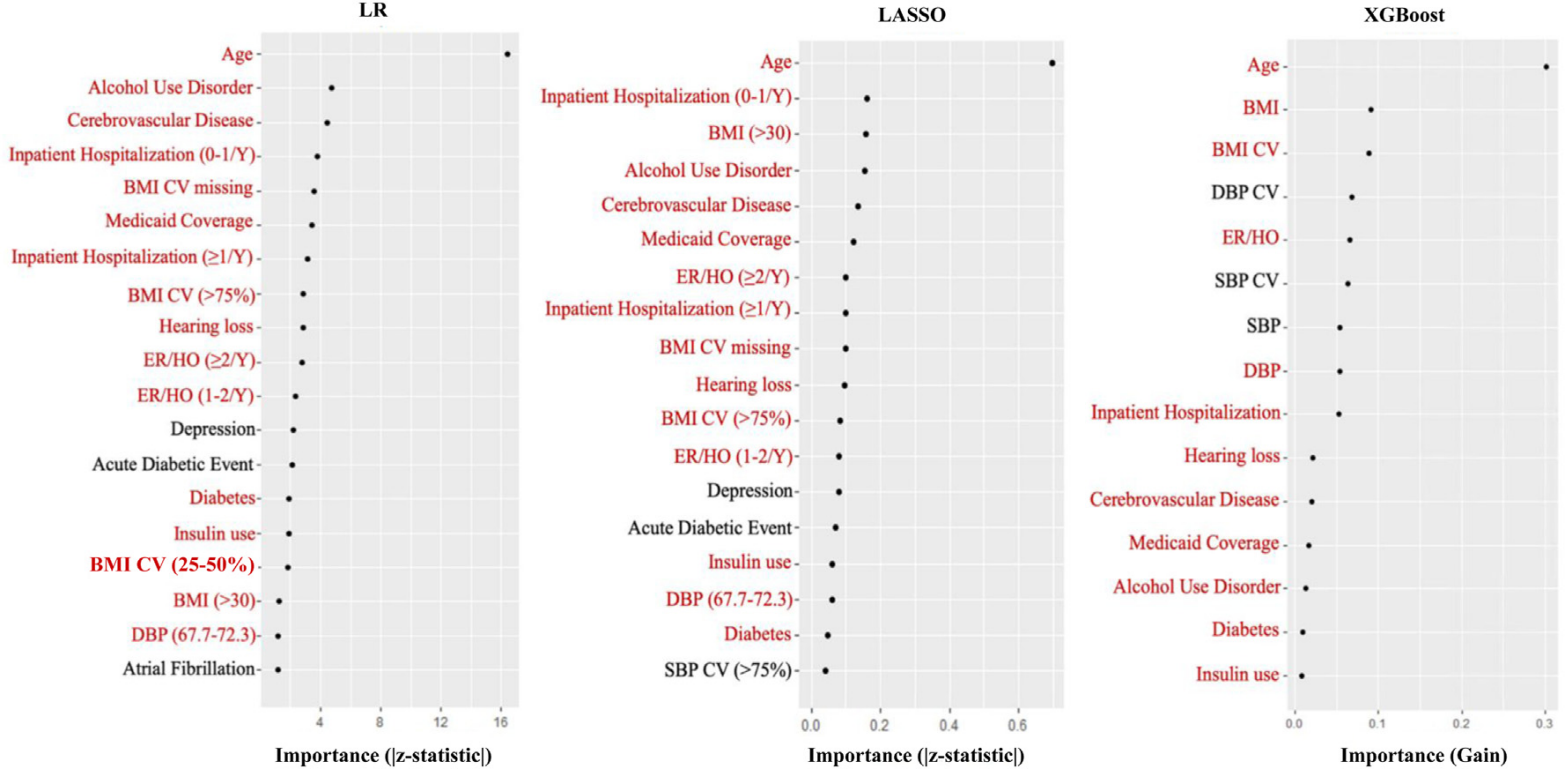
**Model-specific feature importance plots for extended LR, LASSO**^**†**^**, and XGBoost Models predicting the two-year risk of incident all-cause dementia.** Abbreviations: LR: Logistic Regression; LASSO: Least Absolute Shrinkage Operator; XGBoost: Extreme Gradient Boosting; BMI: Body Mass Index; CV: Coefficient of Variation; SBP: Systolic Blood Pressure; DBP: Diastolic Blood Pressure; ER/HO: Emergency Room Visits and Hospital Observations. ^†^All predictors were standardized prior to entry into the LASSO model.

**Fig. 5:**
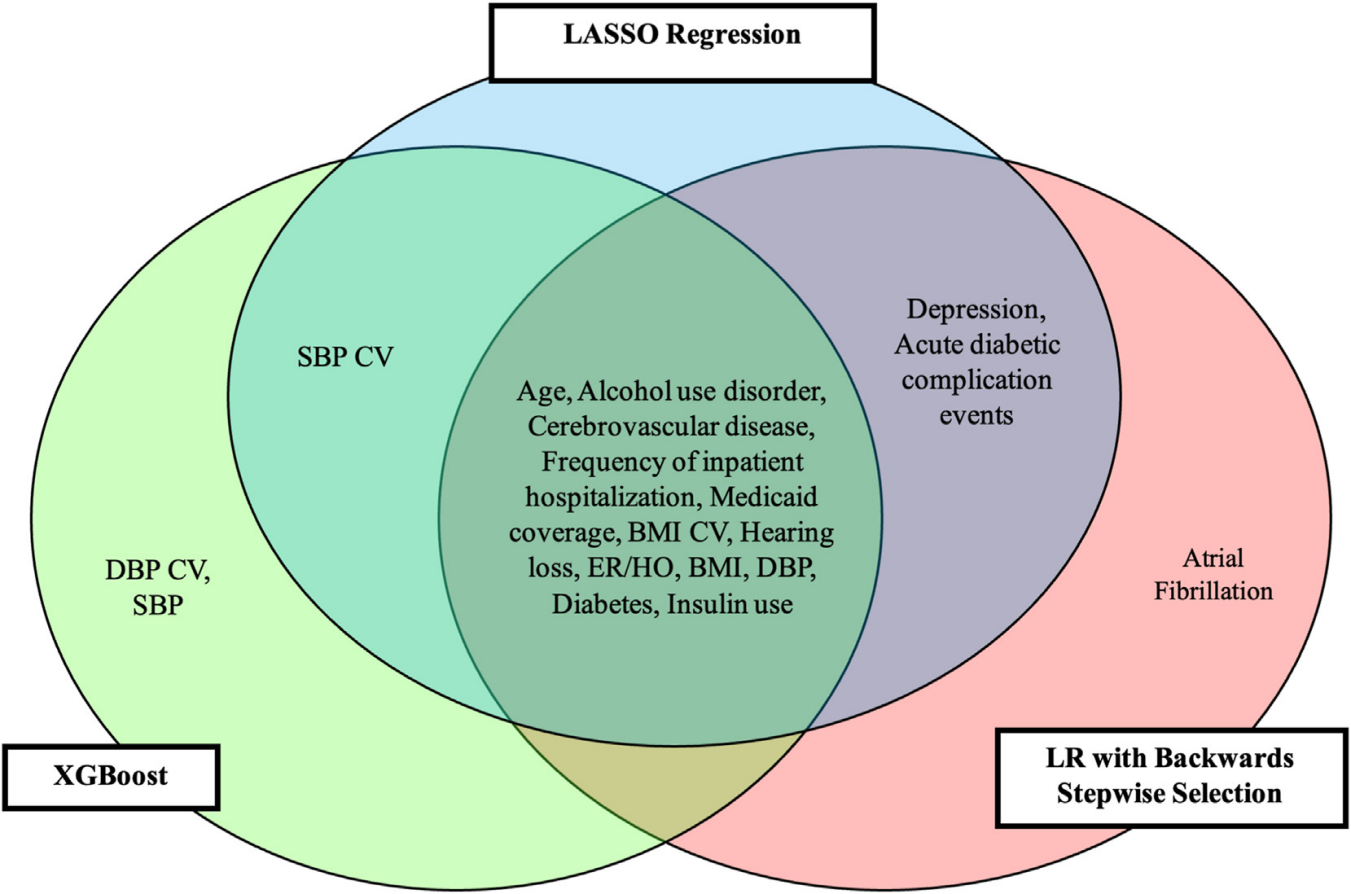
**Venn Diagram comparison of the top 15 predictors for two-year incident, all-cause dementia across XGBoost, LASSO, and LR algorithms.** Abbreviations: LR: Logistic Regression; LASSO: Least Absolute Shrinkage Operator; XGBoost: Extreme Gradient Boosting; BMI: Body Mass Index; CV: Coefficient of Variation; SBP: Systolic Blood Pressure; DBP: Diastolic Blood Pressure; ER/HO: Emergency Room Visits and Hospital Observation.

Fig. 4 shows the feature importance plots for the top-performing models: extended logistic regression, LASSO, and XGBoost, with age identified as the most important predictor across all three models. The top 15 predictors from each model are presented in a Venn diagram (Fig. 5). Of these, 12 predictors (80%) were consistently identified as important across all three models. These include age, alcohol use disorder, cerebrovascular disease, inpatient hospitalizations, Medicaid coverage, BMI coefficient of variation, hearing loss, emergency room visits and hospital observations, BMI, diastolic blood pressure, diabetes, and insulin use. Depression and acute diabetic complication events ranked among the top 15 predictors in both LASSO and logistic regression models, while systolic blood pressure coefficient of variation appeared in the top 15 predictors for both LASSO and XGBoost. Atrial fibrillation was identified as a top predictor only in the logistic regression model, while diastolic blood pressure coefficient of variation and systolic blood pressure were unique to XGBoost.

## Discussion

This study demonstrates that machine learning algorithms utilizing EHR data can effectively predict two-year all-cause dementia risk in AI/AN adults aged 65 years and older who actively use IHS and Tribal health services. The extended logistic regression, LASSO, and XGBoost models each showed strong, and comparable discriminative abilities, with enhanced performance in extended models that incorporated additional health and service use features.

Prediction model development is inherently exploratory, with each algorithm offering distinct strengths and limitations depending on the dataset characteristics. Our findings are consistent with previous research, demonstrating that machine learning models like XGBoost perform similarly to traditional logistic regression in predicting dementia risk using EHR data.^26^^,^^27^

XGBoost offers advanced capabilities, including its ability to handle missing data autonomously and assess complex relationships and interactions, which can streamline the model development process. However, due to the relatively limited set of candidate predictors in this study, the performance improvements from XGBoost were minimal. In future studies with a broader range of predictors, XGBoost could provide greater advantages.

While XGBoost demonstrated comparable performance to simpler models like logistic regression and LASSO, the interpretability and simplicity of logistic regression and LASSO may make them more practical for clinical use. These models provide clear feature weights, which can help clinicians understand and confidently apply the models in real-world settings.

The strong performance of the age-only model highlights age as a primary driver of prediction accuracy, consistent with prior studies.^28^ Reporting the performance of the age-only model provides valuable insights into the influence predictors beyond age alone.

The overlap in top predictors across algorithms reinforces the robustness of the identified dementia risk factors for AI/AN individuals. While these predictors are largely consistent, variations in their rankings across models highlight the different ways algorithms process data. For example, tree-based models, which use metrics like Gain and Gini importance to rank features, tend to prioritize continuous or high-cardinality variables, leading to differences in ranking compared to linear models.^29^ Additionally, LASSO regularization shrinks larger coefficients, effectively selecting a subset of features and refining their relative importance, which results in slightly different rankings compared to logistic regression, which does not use regularization.

To address potential violations of linearity and missing data, continuous predictors (except age) were categorized for LASSO and logistic regression, while XGBoost required no transformation. However, suboptimal categorization can reduce both information and statistical power. A sensitivity analysis using continuous variables for logistic regression and LASSO revealed lower performance, highlighting these models’ limitations in handling non-linear relationships (Supplementary Table S5). This suggests that optimized categorization could enhance performance, whereas XGBoost was able to effectively capture non-linear patterns without requiring transformation.

A novel finding of this investigation is the predictive significance of service utilization during the baseline period. While service utilization is rarely considered a predictor in dementia prediction models, this finding aligns with evidence suggesting that increased hospitalizations and emergency room visits often precede dementia diagnoses.^12^ Incorporating service utilization measures in future models could enhance predictive accuracy and provide valuable insights into early dementia risk.

Cerebrovascular disease also emerged as a top predictor across all models, consistent with prior research linking cerebrovascular disease to dementia risk.^10^ Given the disproportionately high incidence of stroke among AI/AN individuals, this finding highlights the importance of preventive measures targeting cerebrovascular health. Additionally, comorbidities such as depression, diabetes, hearing loss, and BMI variability were significant predictors, reinforcing the need to consider these factors in dementia risk assessments. Medicaid coverage also emerged as an important predictor, potentially reflecting socioeconomic vulnerabilities, as well as varying levels of healthcare access and need.

The strengths of this study include the use of data from a large, geographically diverse cohort of AI/AN older adults. The EHR data source provided longitudinal insights across a broad range of potential risk factors, including health status measurements, medical history, diagnoses, and prescription data. This comprehensive dataset allowed for the consideration of a large number of candidate features. Additionally, the use of a five-year washout/baseline period helped minimize missing data for baseline predictors and assisted in differentiating prevalent and incident dementia cases.

This investigation has several limitations. The low incidence of dementia, combined with the 80%/20% train/test split, reduced the number of cases available for performance evaluation, resulting in wider confidence intervals. Despite this, the performance estimates suggest model robustness with the available data. Additionally, under-detection of dementia diagnoses, particularly from non-IHS and Tribal providers unless Purchased and Referred Care coverage was utilized, may have led to an underestimation of dementia incidence. The observed 3.5% two-year incidence rate in our dataset, which is lower than the 3.1–3.7% one-year incidence reported among AI/AN Medicare beneficiaries,^30^ supports this possibility, though the populations are not directly comparable.

Routinely collected EHR data present inherent limitations for prediction modeling. Key sociodemographic factors, such as education, income, and marital status, were unavailable, which limited the model’s ability to fully account for social determinants of dementia risk. Furthermore, diagnoses of comorbidities and dementia were not externally validated, introducing potential misclassification. The seven-year EHR data window also restricted the ability to capture long-term medical histories, such as prior traumatic brain injury, and constrained the prediction period for dementia.

The IHS is currently funding initiatives aimed at increasing dementia awareness and improving services, including training programs through its Alzheimer’s Disease and Dementia Program to address the needs of patients with dementia. Dementia risk prediction models show promise as valuable tools for IHS and Tribal clinicians, enabling the early identification of high-risk individuals. Future research should focus on translating these models into clinical practice, with an emphasis on establishing operational thresholds that balance sensitivity and specificity to ensure accurate detection while minimizing unnecessary alarm. Additionally, developing practical guidelines for implementation is essential, particularly those that incorporate culturally sensitive approaches tailored to the unique needs of AI/AN communities.

## Contributors

KP and JD researched the data, contributed to the discussion, and wrote the manuscript. KC, MMC, SMM, and JO contributed to the discussion, reviewed and edited the manuscript. LJ designed the study, contributed to the discussion, reviewed and edited the manuscript. LJ is the guarantor of this work and, as such, had full access to all the data in the study and takes responsibility for the integrity of the data and the accuracy of the data analysis.

## Data sharing statement

The data from the IHS Data Project used to support the findings of this study have not been made available for sharing broadly because of IHS and Tribal regulations regarding data confidentiality and security. The research center where Dr. Joan O’Connell, one of the multiple principal investigators (MPIs), works has developed a process to explore opportunities to share project data that would benefit the health of the AI/AN people engaged in the project.

## Declaration of interests

The authors claim no conflict of interests.
