# Supplementary material for: Machine learning to predict dementia for American Indian and Alaska native peoples: a retrospective cohort study
Source: Lancet Reg Health Am. 2025 Feb 13;43:101013. doi: 10.1016/j.lana.2025.101013 (PMC11875197; doi:10.1016/j.lana.2025.101013)
Supplement: Supplementary Methods and Tables S1–S5 [file mmc1.docx]

**Machine learning to predict dementia for American Indian and Alaska Native Peoples: a retrospective cohort study**

**Supplementary Materials**

**Table of Contents**

**Supplemental Methods……………………………………………………………………………………………… 2**

**Supplemental Methods References…………………………………………………………………………………. 3**

**Supplementary Table 1……………………………………………………………………………………………….4**

**Supplementary Table 2……………………………………………………………………………………………….5**

**Supplementary Table 3……………………………………………………………………………………………….7**

**Supplementary Table 4……………………………………………………………………………………………….8**

**Supplementary Table 5……………………………………………………………………………………………….9**

**Supplemental Methods**

**Logistic Regression with Backward Stepwise Selection**

Logistic regression is a traditional statistical method that models the probability of an outcome by representing the log-odds as a linear combination of predictors.^1^ Key assumptions for logistic regression include a linear relationship between each predictor and the log-odds of the outcome, sufficient sample size, defined as at least 10 outcome events per predictor, minimal multicollinearity, and the resolution of data missingness prior to model development. ^2^

Backward Stepwise Selection is a feature selection method for logistic regression that starts with all predictors and removing predictors in a stepwise fashion have the least impact on model fit based on Akaike information criterion (AIC). This method aids in creating a more parsimonious model when handling a large number of predictors, though it has its limitations. Logistic regression with Backward Stepwise Selection can lead to unstable models, as the sequence of predictor removal and the initial pool of predictors can affect the final model. Additionally, model coefficients and p-values may be downwardly biased, and, without regularization, models are susceptible to overfitting.^3-6^ The glmnet package and Mass package were utilized for logistic regression model development with Backward Stepwise Selection in R software.^6-8^

**Least Absolute Shrinkage and Selection Operator (LASSO)**

LASSO (Least Absolute Shrinkage and Selection Operator) regression is an L1 regularization method that can be applied to regression problems, both linear and logistic. It performs automatic feature selection by shrinking the coefficients of less relevant predictors to zero, effectively excluding them from the model. This regularization helps prevent overfitting and improves model generalizability, especially when there are many predictors, of which only a few are expected to be strongly predictive.^9^ When LASSO is applied to a logisitic regression problem, the underlying assumption of the linear relationship of each predictor to the log-odds of the outcome is maintained. Additionally, LASSO has limitations with highly correlated predictors, often selecting only one predictor from a correlated set.^10^ The *glmnet* package and R software were used for LASSO prediction model development.^6,7^

**Random Forest**

Random Forest is a popular decision-tree based machine learning algorithm. In random forest, data is first sampled with replacement (bootstrap sampling) to create multiple datasets. Random Forest generates numerous decision trees by bootstrapping the data (sampling with replacement) and using a random subset of variables at each split, creating a diverse set of trees. Predictions are then aggregated (bagging) across all trees, making Random Forest more accurate and adaptable than individual trees.^11,12^ Strengths of Random Forest include its ability to handle numerous predictors, capture complex interactions, work well with correlated variables, and model non-linear relationships without needing to specify an underlying data model.^11,12^ However, Random Forest can be challenging to interpret, as the large number of trees can obscure individual predictor influence. Variable importance measures, such as those based on the Gini coefficient, are used to assess predictor impact, although Random Forest may bias towards predictors with more categories or higher measurement scales.^12^ In this investigation, the parsnip package from the tidymodels framework was employed to implement the random forest model, specifying the ranger engine, all within the R software environment.^8,13-15^

**XGBoost (Extreme Gradient Boosting)**

XGBoost, an advanced implementation of the gradient-boosted decision trees, is a ML algorithm that utilizes boosting to sequentially create models that adjust for errors made in previous models until no further corrections can be made.^16^ XGBoost was selected for this investigation for its strengths including the ability to capture non-linear relationships, built in regularization, robustness for handling collinearity and sparse features, novel and intelligent approach to missing data (the sparsity-aware split finding algorithm), parallel and distributed computing for fast and efficient learning, and success in ML competitions.^17^  However, XGBoost’s complexity can hinder interpretability, especially regarding predictor interactions.^18^ The *xgboost* package and R software were used for XGBoost model development.^6,18^

**Supplemental Methods References**

1. Schreiber-Gregory D, Bader K. Logistic and linear regression assumptions: Violation recognition and control. Henry M Jackson Foundation. 2018;**247**:22. Available at: <https://www.lexjansen.com/pharmasug/2019/ST/PharmaSUG-2019-ST-058.pdf> (assessed October 3, 2021).

2. Ottenbacher KJ, Ottenbacher HR, Tooth L, Ostir GV. A review of two journals found that articles using multivariable logistic regression frequently did not report commonly recommended assumptions. *J Clin Epidemiol*. 2004;**57**:1147–52.

3. Peduzzi P, Concato J, Kemper E, Holford TR, Feinstein AR. A simulation study of the number of events per variable in logistic regression analysis. *J Clin Epidemiol*. 1996;**49:**1373–9.

4. Morozova O, Levina O, Uusküla A, Heimer R. Comparison of subset selection methods in linear regression in the context of health-related quality of life and substance abuse in Russia. *BMC Med Res Methodol*. 2015;**15**:1–7. https://doi.org/10.1186/s12874-015-0066-2.

5. Derksen S, Keselman HJ. Backward, forward and stepwise automated subset selection algorithms: Frequency of obtaining authentic and noise variables. Br J Math Stat Psychol. 1992;**45**:265–82.

6. Friedman J, Hastie T, Tibshirani R. Regularization paths for generalized linear models via coordinate descent. *J Stat Softw*. 2010;**33**:1–22. https://doi.org/10.18637/jss.v033.i01.

7. Venables, W.N. and Ripley, B.D. (2002) Modern Applied Statistics with S. Springer, New York, 271–300. <https://doi.org/10.1007/978-0-387-21706-2>

8. R Core Team. R: a language and environment for statistical computing. R Foundation for Staistical Computing, Vienna; Austria, 2023. Available at: <https://www.r-project.org/>.

9. Tibshirani R. Regression shrinkage and selection via the lasso*. J R Stat Soc Series B Stat Methodol*. 1996; **58**:267–88.

10. Signorino CS, Kirchner A. Using LASSO to model interactions and nonlinearities in survey data. *Surv Pract*. 2018;**11**:1–0. https://doi.org/10.29115/SP-2018-0005.

11. Rigatti SJ. Random forest. *J. Insur. Med*. 2017;**47**:31–9. https://doi.org/10.17849/insm-47-01-31-39.1.

12. Boulesteix AL, Janitza S, Kruppa J, König IR. Overview of random forest methodology and practical guidance with emphasis on computational biology and bioinformatics. *Wiley Interdiscip Rev Data Min Knowl Discov*. 2012;**2**:493–507. https://doi.org/10.1002/widm.1072.

13. Kuhn M, Vaughan D. parsnip: A Common API to Modeling and Analysis Functions. R package version 1.2.1, 2024. Available at: <https://parsnip.tidymodels.org/>.

14. Kuhn M, Wickham H. Tidymodels: a collection of packages for modeling and machine learning using tidyverse principles. 2020. Available at: <https://tidymodels.tidymodels.org/>.

15. Wright, M. N. & Ziegler, A. (2017). ranger: A fast implementation of random forests for high dimensional data in C++ and R. *J Stat Softw*. **77**:1–17. <https://doi.org/10.18637/jss.v077.i01>.

16. Friedman JH. Greedy function approximation: a gradient boosting machine. *Ann Stat*. 2001:1189–232.

17. Chen T, Guestrin C. Xgboost: A scalable tree boosting system. In Proceedings of the 22nd acm sigkdd international conference on knowledge discovery and data mining 2016 Aug 13 (pp. 785–794). https://doi.org/10.1145/2939672.2939785.

18. Waljee AK, Higgins PD. Machine learning in medicine: a primer for physicians*. Am J Gastroenterol*. 2010;**105**:1224–6.

**Supplementary Table 1. ICD-9 Codes and Related Terms for Identifying Predictors**

|  | **ICD-9 Diagnostic Codes** | **Diagnosis Term** |
| --- | --- | --- |
| **Hypoglycemia** |  |  |
|  | 251.0 | Hypoglycemic Coma |
|  | 251.1 | Other Specified Hypoglycemia |
|  | 251.2 | Hypoglycemia, Unspecified |
|  | 962.3 | Poisoning by Insulins or Antidiabetic Agents |
|  | 250.8* | Diabetes with other manifestations (diabetic hypoglycemia NOS or hypoglycemia shock NOS) |
| *Excluding admissions with the following secondary ICD-9 codes |  |  |
|  | 259.8 | Secondary diabetic glycogenosis |
|  | 272.7 | Diabetic lipidosis |
|  | 681.xx, 682.xx, 686.9x | Cellulitis |
|  | 707.1-707.9 | Ulcers of the lower extremity |
|  | 709.3 | Oppenheim-Urbach syndrome |
|  | 730.0-730.2, 731.8 | Osteomyelitis |
| **Hyperglycemia** |  |  |
|  | 250.02 | Diabetes type 2 without complications, uncontrolled |
|  | 250.03 | Diabetes type 1 without complications, uncontrolled |
|  | 250.1 | Diabetic ketoacidosis |
|  | 250.2 | Diabetes with hypersomolarity |
|  | 250.3 | Diatebetes with other coma (diabetic coma with ketoacidosis, diabetic hypoglycemic coma, insulin coma NOS) |
| **Diabetic Ketoacidosis** |  |  |
|  | 250.10 | Diabetic Ketoacidosis, type 2 or unspecified type, not stated as uncontrolled |
|  | 250.11 | Diabetic Ketoacidosis, type 1, not stated as uncontrolled |
|  | 250.12 | Diabetic Ketoacidosis, type 2 or unspecified type, uncontrolled |
|  | 250.13 | Diabetic Ketoacidosis, type 1, uncontrolled |
| Excluding admissions with the following secondary ICD-9 code: | 250.3 | Diabetes with other coma |
| **Stroke^1^** |  |  |
|  | 362.3, 433.x1, 434.x1, 436 | Acute ischemic stroke |
|  | 431.x | Intracerebral hemorrhage |
|  | 430.x | Subarachnoid hemorrhage |
|  | 435.x | Transient ischemic attack |
| **Traumatic Brain Injury^2^** |  |  |
|  | 800.0, 800.5, 801.0, 801.5, 803.0, 803.5, 804.0, 804.5 | Skull Fracture |
|  | 850.0, 850.1, 850.5, 850.9 | Concussion |
|  | 854.0 | Intracranial injury of other and unspecified nature |
|  | 959.01 | Head injury, unspecified |

1. ICD-9 code list adapted from: Kokotailo RA, Hill MD. Coding of stroke and stroke risk factors using international classification of diseases, revisions 9 and 10. *Stroke.* 2005;**36**:1776-81.

2. ICD-9 code list for Traumatic Brain Injury adapted from: Bazarian JJ, Veazie P, Mookerjee S, Lerner EB. Accuracy of mild traumatic brain injury case ascertainment using ICD‐9 codes. *Acad Emerg Med*. 2006;**13**:31-8.

**Supplementary Table 2. Baseline Clinical and Demographic Characteristics of Included vs. Excluded Patients Based on Inclusion/Exclusion Criteria**

|  | **Included Patients**  **N=17,398**  **N (%)** | **Excluded Patients**  **N=20,746**  **N (%)** |
| --- | --- | --- |
| **Age (years)** |  |  |
| Mean (SD) | 73.1 (6.3) | 75.3 (8.0) |
| Range | (65.0-103.0) | (61.0-110.3) |
| **Sex** |  |  |
| Female | 10,409 (59.8) | 11,260 (54.3) |
| **Insurance Coverage** |  |  |
| Private | 2,942 (16.9) | 3,142 (15.2) |
| Medicaid | 2,354 (13.5) | 2,628 (12.7) |
| Medicare | 16,959 (97.5) | 9,758 (47.0) |
| Medicare Coverage Status Unknown/Missing | 0 (0.0) | 9,994 (48.2) |
| None | 346 (2.0) | 2,245 (10.8) |
| **Body Mass Index (BMI)**^*^ |  |  |
| Underweight | 113 (0.7) | 259 (1.3) |
| Normal Weight | 3,015 (17.3) | 2,389 (11.5) |
| Overweight | 5,952 (34.2) | 3,185 (15.4) |
| Obese | 7,358 (42.3) | 3,573 (17.2) |
| Missing | 960 (5.5) | 11,340 (54.7) |
| **Blood Pressure**^†^ |  |  |
| Normal | 1,598 (9.2) | 1,708 (8.2) |
| Elevated | 4,040 (23.2) | 2,421 (11.7) |
| High | 11,484 (66.0) | 7,176 (34.6) |
| Missing | 276 (1.6) | 9,441 (45.5) |
| **Cardiovascular Disease Subtype**^‡^ |  |  |
| Cerebrovascular Disease | 2,069 (11.9) | 1,452 (7.0) |
| Ischemic Heart Disease | 4,560 (26.2) | 3,694 (17.8) |
| Vascular Disease | 5,196 (29.9) | 3,483 (16.8) |
| Congestive Heart Failure/Other | 6,462 (37.1) | 4,578 (22.1) |
| **Cardiovascular Conditions/Events**^‡^ |  |  |
| Stroke or Ischemic Attack | 1,121 (6.4) | 768 (3.7) |
| Atrial Fibrillation | 1,169 (6.7) | 904 (4.4) |
| **Other Comorbidities**^‡^ |  |  |
| Diabetes | 8,264 (47.5) | 4,924 (23.7) |
| Hypertension | 14,829 (85.2) | 10,032 (48.4) |
| Cancer (All) | 1,729 (9.9) | 1,765 (8.5) |
| Chronic Kidney Disease | 3,384 (19.5) | 2,402 (11.6) |
| Traumatic Brain Injury | 442 (2.5) | 243 (1.2) |
| Hearing Loss | 2,844 (16.4) | 1,319 (6.4) |
| **Mental Health Diagnoses**^‡^ |  |  |
| Depression | 3,794 (21.8) | 2,304 (11.1) |
| Mood/Anxiety Disorder (Excluding Depression) | 959 (5.5) | 534 (2.6) |
| **Substance Use Disorder**^‡^ |  |  |
| Alcohol Use Disorder | 862 (5.0) | 685 (3.3) |
| Tobacco Use Disorder | 2,619 (15.1) | 1,788 (8.6) |
| **Antidiabetic Medications** |  |  |
| Metformin | 5,154 (29.6) | 1,962 (9.5) |
| Sulfonylureas | 4,052 (23.3) | 1,881 (9.1) |
| Thiazolidinediones | 3,426 (19.7) | 1,346 (6.5) |
| Insulin | 2,752 (15.8) | 1,627 (7.8) |
| **Medications for Hypertension^§^** | 14,714 (84.6) | 8,482 (40.9) |
| **Medications for Cardiovascular Disease**^¶^ | 11,030 (63.4) | 5,100 (24.6) |
| **Average Inpatient Hospitalizations per Fiscal Year** |  |  |
| 0 | 12,808 (73.6) | 16,403 (79.1) |
| >0 to <1 | 4,171 (24.0) | 2,215 (10.7) |
| ≥1 | 419 (2.4) | 1,162 (5.6) |
| **Average Emergency Room Visits or Hospital Observations per Fiscal Year** |  |  |
| <1 | 14,086 (81.0) | 18,348 (88.4) |
| ≥1 to <2 | 2,103 (12.1) | 1,326 (6.4) |
| ≥ 2 | 1,209 (7.0) | 1,072 (5.2) |
|  |  |  |
| **Severe Acute Diabetic Event** | 604 (3.5) | 322 (1.6) |

^*^Average BMI across the baseline period with WHO algorithm utilized for BMI categorization. Underweight: BMI < 18.5; Normal Weight: BMI 18.5 to 25; Overweight: 25 < BMI < 30; Obese: BMI ≥ 30

^†^Average blood pressure across the baseline period with the following definitions: Normal: Systolic Blood Pressure (SBP) < 120 mmHg and Diastolic Blood Pressure (DBP) < 80 mmHg; Elevated: 120 mmHg ≤ SBP < 130 mmHg and DBP < 80 mmHg; High: SBP ≥130 mmHg or DBP ≥ 80 mmHg

^‡^Comorbidities present in electronic health records during the baseline period

^§^Anti-hypertensive medications included diuretics, beta blockers, calcium channel blockers, angiotensin converting enzyme inhibitors, angiotensin receptor blockers, and other anti-hypertension medications

^¶^Cardiovascular disease medications included statins and other antihyperlipidemic medications

**Supplementary Table 3. Optimized hyper-parameter values for each model using grid-search**

| **Hyperparameter** | **LR**  **(DRS-based)** | **LR**  **(Extended)** | **LASSO**  **(DRS-based)** | **LASSO**  **(Extended)** | **RF**  **(DRS-based)** | **RF**  **(Extended)** | **XGBoost**  **(DRS-based)** | **XGBoost**  **(Extended)** |
| --- | --- | --- | --- | --- | --- | --- | --- | --- |
| lambda | .. | .. | 0.00087 | 0.00139 | .. | .. | .. | .. |
| m-try | .. | .. | .. | .. | 2 | 10 | 3 | 8 |
| min-n | .. | .. | .. | .. | 10 | 10 | 1610 | 1610 |
| trees | .. | .. | .. | .. | .. | .. | 12 | 12 |
| tree-depth | .. | .. | .. | .. | .. | .. | 12 | 12 |
| learn rate | .. | .. | .. | .. | .. | .. | 0.00393 | 0.00393 |
| loss reduction | .. | .. | .. | .. | .. | .. | 3.63724e-9 | 3.63724e-9 |
| sample size | .. | .. | .. | .. | .. | .. | 0.4478 | 0.4478 |

Abbreviations: LR: Logistic Regression; LASSO: Least Absolute Shrinkage Operator; RF: Random Forest; XGBoost: Extreme Gradient Boosting; DRS: Dementia Risk Score

**Supplementary Table 4: Model performance, measured by area under the receiver operator characteristic curve (AUC), on testing data for one-year incident dementia prediction**

| **Model** | **AUC (95% CI)** |
| --- | --- |
| LR (Age-Only) | 0.69 (0.62, 0.77) |
| LR (DRS-Based) | 0.74 (0.68, 0.80) |
| LR (Extended) | 0.79 (0.74, 0.84) |
| LASSO (DRS-Based) | 0.74 (0.67, 0.80) |
| LASSO (Extended) | 0.79 (0.74, 0.84) |
| RF (DRS-Based) | 0.74 (0.68, 0.81) |
| RF (Extended) | 0.76 (0.73, 0.84) |
| XGBoost (DRS-Based) | 0.72 (0.63, 0.77) |
| XGBoost (Extended) | 0.80 (0.73, 0.83) |

Abbreviations: LR: Logistic Regression; LASSO: Least Absolute Shrinkage Operator; RF: Random Forest; XGBoost: Extreme Gradient Boosting; DRS: Dementia Risk Score; CI: Confidence Interval

**Supplementary Table 5. Model performance (AUC, 95% CI) with continuous vs. categorized predictors**

| **Model** | **Continuous Predictors**  **(AUC, 95% CI)** | **Categorical Predictors**  **(AUC, 95% CI)** |
| --- | --- | --- |
| LR (DRS-Based) | 0.80 (0.77, 0.84) | 0.80 (0.77, 0.83) |
| LR (Extended) | 0.83 (0.79, 0.86) | 0.83 (0.79, 0.86) |
| LASSO (DRS-Based) | 0.80 (0.76, 0.83) | 0.80 (0.77, 0.83) |
| LASSO (Extended) | 0.82 (0.78, 0.85) | 0.83 (0.79, 0.86) |

Abbreviations: LR: Logistic Regression; LASSO: Least Absolute Shrinkage Operator; DRS: Dementia Risk Score; AUC: Area Under the Receiver Operating Characteristic Curve; CI: Confidence Interval
